# 10-YEAR ANNIVERSARY OF THE SLEEP HEALTH PERSPECTIVE: INSIGHTS FOR AGING RESEARCHERS

**DOI:** 10.1093/geroni/igae098.0846

**Published:** 2024-12-31

**Authors:** Soomi Lee, Christopher Kaufmann, Daniel Buysse

**Affiliations:** Chair; Co-Chair; Discussant

## Abstract

In 2014, Dr. Buysse published a seminal paper, titled “Sleep health: can we define it? Does it matter?” which proposed that sleep health extends beyond the absence of sleep disorders. He advocated for a preventive approach where individuals must address multiple aspects of sleep to support optimal functioning and health. The concept of sleep health initiated a paradigm shift, prompting researchers to consider multiple sleep variables, not solely sleep duration, to gain a holistic understanding of individuals’ sleep patterns. As we approach the 10-year anniversary of the sleep health perspective, this symposium brings together four esteemed speakers to discuss methodological approaches and empirical findings related to the link between sleep health and cardiovascular and cognitive aging outcomes, as well as social disparities in sleep health. Dr. Wallace will outline methodological challenges in assessing sleep health and clarify the advantages and disadvantages of various methods. Dr. Buxton will present a multi-dimensional sleep health model assessed by actigraphy and its association with cardiometabolic and cognitive aging outcomes in two US midlife and older adult samples. Dr. Knutson will share findings from US and Brazil population-based studies with subjective and objective dimensions of sleep health, including gender and racial differences in sleep health and cardiovascular risks. Dr. Stone will integrate epidemiology and clinical evidence to explore the link between sleep health, cognitive decline, and the onset of dementia. Finally, the discussant, Dr. Buysse, will synthesize the significance of findings, and identify opportunities for extending the sleep health perspective to future research and opportunities. Sleep, Circadian Rhythms and Aging Interest Group Sponsored Symposium

